# Investigation on performance of particle swarm optimization (PSO) algorithm based fuzzy inference system (PSOFIS) in a combination of CFD modeling for prediction of fluid flow

**DOI:** 10.1038/s41598-021-81111-z

**Published:** 2021-01-15

**Authors:** Meisam Babanezhad, Iman Behroyan, Ali Taghvaie Nakhjiri, Azam Marjani, Mashallah Rezakazemi, Amir Heydarinasab, Saeed Shirazian

**Affiliations:** 1 Institute of Research and Development, Duy Tan University, Da Nang, 550000 Vietnam; 2 Faculty of Electrical – Electronic Engineering, Duy Tan University, Da Nang, 550000 Vietnam; 3 Department of Artificial Intelligence, Shunderman Industrial Strategy Co., Tehran, Iran; 4 Faculty of Mechanical and Energy Engineering, Shahid Beheshti University, Tehran, Iran; 5 Department of Computational Fluid Dynamics, Shunderman Industrial Strategy Co., Tehran, Iran; 6 Department of Petroleum and Chemical Engineering, Science and Research Branch, Islamic Azad University, Tehran, Iran; 7 Department for Management of Science and Technology Development, Ton Duc Thang University, Ho Chi Minh City, Vietnam; 8 Faculty of Applied Sciences, Ton Duc Thang University, Ho Chi Minh City, Vietnam; 9 Faculty of Chemical and Materials Engineering, Shahrood University of Technology, Shahrood, Iran; 10 Laboratory of Computational Modeling of Drugs, South Ural State University, 76 Lenin prospekt, Chelyabinsk, Russia 454080

**Keywords:** Chemical engineering, Mechanical engineering, Computational science

## Abstract

Herein, a reactor of bubble column type with non-equilibrium thermal condition between air and water is mechanistically modeled and simulated by the CFD technique. Moreover, the combination of the adaptive network (AN) trainer with the fuzzy inference system (FIS) as the artificial intelligence method calling ANFIS has already shown potential in the optimization of CFD approach. Although the artificial intelligence method of particle swarm optimization (PSO) algorithm based fuzzy inference system (PSOFIS) has a good background for optimizing the other fields of research, there are not any investigations on the cooperation of this method with the CFD. The PSOFIS can reduce all the difficulties and simplify the investigation by elimination of the additional CFD simulations. In fact, after achieving the best intelligence, all the predictions can be done by the PSOFIS instead of the massive computational efforts needed for CFD modeling. The first aim of this study is to develop the PSOFIS for use in the CFD approach application. The second one is to make a comparison between the PSOFIS and ANFIS for the accurate prediction of the CFD results. In the present study, the CFD data are learned by the PSOFIS for prediction of the water velocity inside the bubble column. The values of input numbers, swarm sizes, and inertia weights are investigated for the best intelligence. Once the best intelligence is achieved, there is no need to mesh refinement in the CFD domain. The mesh density can be increased, and the newer predictions can be done in an easier way by the PSOFIS with much less computational efforts. For a strong verification, the results of the PSOFIS in the prediction of the liquid velocity are compared with those of the ANFIS. It was shown that for the same fuzzy set parameters, the PSOFIS predictions are closer to the CFD in comparison with the ANFIS. The regression number (R) of the PSOFIS (0.98) was a little more than that of the ANFIS (0.97). The PSOFIS showed a powerful potential in mesh density increment from 9477 to 774,468 and accurate predictions for the new nodes independent of the CFD modeling.

## Introduction

Various sorts of reactors have been designed and exploited for multi-phase reactions, among which bubble column reactor types have attracted a huge deal of interest within the environmental, biopharmaceuticals, petrochemical, wastewater treatment, etc.^1–5^. These versatile chemical/biochemical reactors could bring advantages as a result of decent heat and mass transfers, easy operation, and so on^6,7^. There are numerous studies focusing on the gas–water systems for assessing hydrodynamic performance of these reactors^8,9^ and it is of vital importance to create a simulation and assess the fluid flow parameters inside the bubble columns for better process understanding. Model-based process development approach can be utilized in this context for process intensification and improvements. The findings could considerably help the engineers and researchers in design, optimization, operation, etc.^1,10^.

Computational fluid dynamics (CFD)^11^ is a precise approach to study two-phase fluid flow in the bubble columns, and analyze the interactions between phases, i.e. gas and liquid. CFD simulations have been utilized in various studies^12–14^ to assess these types of reactors containing water and air systems. Due to the presence of two phases inside the reactor and interaction between them, almost all studies considered the Eulerian CFD model and just the mass transfer in the reactor both internally and between phases^7,15–17^. According to literature, there are no CFD studies considering non-equilibrium thermal conditions between air and water in a bubble column reactor. This case study is considered, for the first time, in the present paper. The temperature difference between air and water adds the energy governing equations to the CFD modeling to build the comprehensive simulation methodology. Considering the interphase heat transfer between gas and liquid makes CFD modeling of the reactor more complicated, and sophisticated methods are required.

Besides, according to the literature, artificial intelligence (AI) methods are helpful ways for enhancement of applications of the CFD modeling^18–22^. A few studies have already reported the usage of ANFIS model with the CFD for the prediction of fluid flow characteristics in various circumstances^23–28^. Although the PSOFIS method has been already investigated for the data optimizations in many engineering aspects^29–32^, there are no investigations adopted the PSOFIS in cooperation with the CFD modeling. For example, Shi and Eberhart^29^ reported the potential of the PSOFIS by benchmarking the experimental data. Hu et al.^30^ performed the PSOFIS to minimize the power loss in electricity distribution systems. We investigated the effects of parameters of the PSOFIS on the best intelligence in detail. Therefore, the AI method of particle swarm optimization (PSO) algorithm based fuzzy inference system (PSOFIS) is selected in this work to help the CFD modeling. In order to achieve the most accurate prediction of the algorithm, the values of input numbers, swarm sizes, and inertia weights are investigated. The increase of the mesh density is also tested for the first time by the PSOFIS. An additional comparison is made between PSOFIS and ANFIS results regarding the accuracy of the methods.

## Methodology

### Geometry of the reactor

A cylindrical column is considered with a diameter of 29 cm and a length of 2 m here for the computational tasks and understanding the process. In this reactor, the existing gas phase (air) is sent to the column of water from the bottom. Air velocity and air temperature are respectively 0.02 m/s and 400 K, while the water temperature is 295 K.

### CFD approach

The Eulerian–Eulerian two-phase model was utilized in this work with the two-equation standard $k - \varepsilon$ turbulence model. In this fluid modeling approach, the following equations are derived for each phase inside the reactor^12^:

- Continuity equation of phase *k*:
  1 $$\frac{\partial }{\partial t}\left( { \in_{k} \rho_{k} } \right) + \frac{\partial }{{\partial x_{i} }}\left( { \in_{k} \rho_{k} u_{k,i} } \right) = 0.$$
- Momentum equation of phase *k*:
  2 $$\frac{\partial }{\partial t}\left( { \in_{k} \rho_{k} u_{k,i} } \right) + u_{j} \frac{\partial }{{\partial x_{j} }}\left( { \in_{k} \rho_{k} u_{k,i} } \right) = - \in_{k} \frac{\partial P}{{\partial x_{i} }} + \in_{k} \rho_{k} g + \frac{\partial }{{\partial x_{j} }}\left( { \in_{k} \left( {\mu + \mu_{t} } \right)\frac{{\partial u_{k,i} }}{{\partial x_{j} }}} \right) + F_{I}$$

The energy equation is used to calculate the interphase heat transfer between air and water^33^.

Energy equation of phase *k*:

3 $$\frac{{\partial \rho_{k} \in_{k} H_{k} }}{\partial t} + \nabla .\left( {\rho_{k} \in_{k} u_{k,i} H_{k} } \right) = \nabla \left[ { \in_{k} \left( {k + k_{t} } \right)\left( {\nabla T_{k} } \right)} \right] + Q_{I} .$$

The momentum interphase interactions are the summation of the drag force and the turbulent dispersion defining as follows:

4 $$\begin{gathered} F_{I} = F_{td} + F_{d} \hfill \\ F_{td} = - C_{td} \rho_{water} k\nabla \in_{water} , \hfill \\ \end{gathered}$$

where *k* and *C*_*td*_ are the water turbulent kinetic energy per unit of mass and turbulent dispersion coefficient, respectively. The value of 0.3 is considered for the turbulent dispersion coefficient based on the study of^22^.

5 $$F_{d} = \frac{1}{8}C_{D} a_{if} \rho_{k} \left| {\overrightarrow {{u_{k,i} }} - \overrightarrow {{u_{i,k} }} } \right|\left( {\overrightarrow {{u_{k,i} }} - \overrightarrow {{u_{i,k} }} } \right) ,$$

6 $$Q_{I} = ha_{if} \left( {T_{k,i} - T_{i,k} } \right).$$

The Schiller-Naumann^34,35^ drag coefficient (*C*_*D*_) is adopted for inter-phase interaction of the momentum equations, while for interphase heat transfer coefficient (*h*) between air bubble and water, the Ranz-Marshall^36^ equation is used. It should be noted that the air is considered as incompressible fluid and the bubble shape is supposed to be spherical. So, the interphase area (*a*_*if*_) is given as follows:

7 $$a_{if} = \frac{{6 \in_{air} }}{{d_{air \,bubble} }}.$$

In this study, the standard $k - \varepsilon$ turbulence model was selected. The key mathematical models utilized in the present work taken from the literature^37–42^:

8 $$\frac{\partial }{\partial t}\left( { \in_{k} \rho_{k} k_{k} } \right) + \nabla \cdot \left( { \in_{k} \rho_{k} u_{k,i} k_{k} } \right) = \nabla \cdot \left[ {\left( {\frac{{\mu_{k}^{t} }}{{\sigma_{k} }}} \right)\nabla \left( {k_{k} } \right)} \right] + G_{k} - \rho_{k} \varepsilon_{k} ,$$

9 $$\frac{\partial }{\partial t}\left( { \in_{k} \rho_{k} \varepsilon_{k} } \right) + \nabla \cdot \left( {\rho_{k} \varepsilon_{k} u_{k,i} } \right) = \nabla \cdot \left[ {\frac{{\mu_{k}^{t} }}{{\sigma_{\varepsilon } }}\nabla \varepsilon_{k} } \right] + \frac{{\varepsilon_{k} }}{{k_{k} }}\left( {C_{1\varepsilon } G_{k} - C_{2\varepsilon } \rho_{k} \varepsilon_{k} } \right),$$

10 $$G_{k} = \mu_{k}^{t} (\nabla u_{k,i} + \left( {\nabla u_{k,i} )^{T} } \right),$$

11 $$\mu_{k}^{t} = \rho_{k} C_{\mu } \frac{{k^{2} }}{\varepsilon }.$$

### Grid partition test and validation

**Figure 1:**
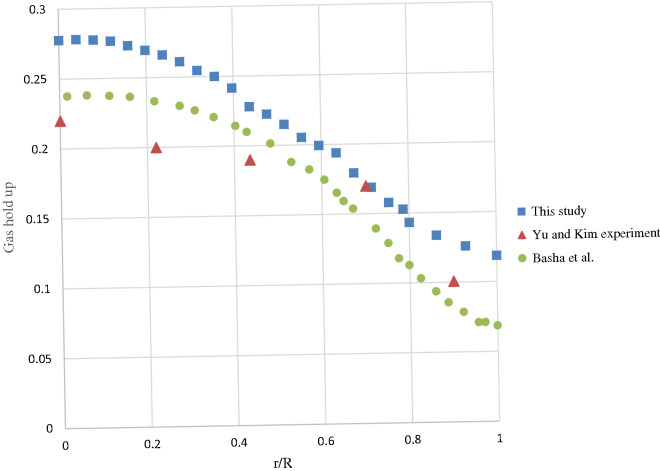
Validation of present CFD study versus Yu and Kim experiment and numerical investigation of Basha et al.

F the grid independency test process, two mesh arrangements are employed for the simulation of the reactor: the former with 9477 nodes and the latter with 18,954 nodes. The CFD results have been compared in the case of the water and air velocities and the deviation was less than 0.04%. For less computational efforts, the first mesh density has been adopted in this study. For verification of the CFD simulation, the results of gas hold-up predictions are compared with the experiment of Yu and Kim^43^ and the numerical results of Basha et al.^34^ (Fig. 1).

### Particle swarm optimization

**Figure 2:**
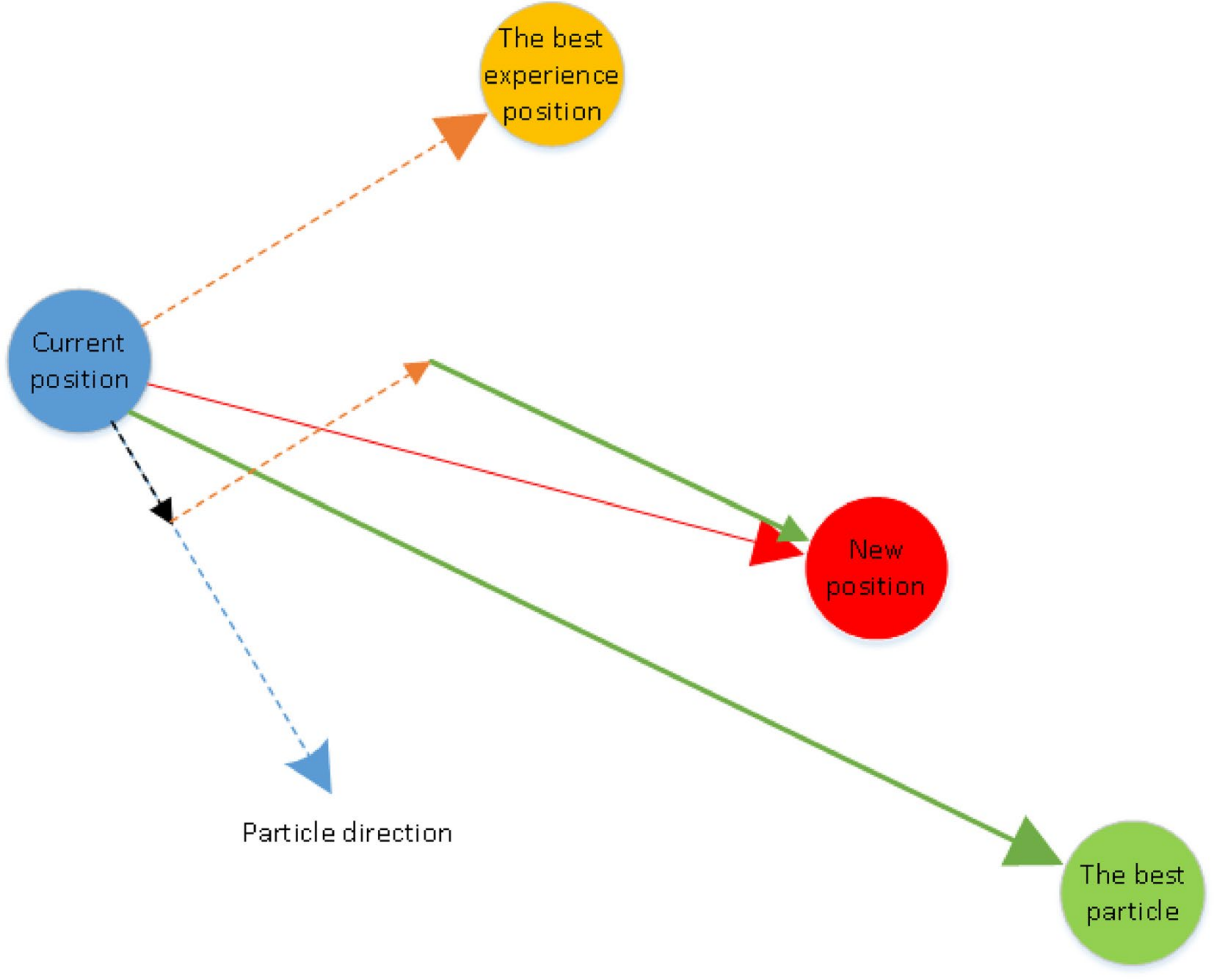
Schematics of PSO algorithm.

Particle swarm optimization (PSO) is population-based algorithms that generate and use random variables. This algorithm has been spired by the collective behavior of animals like the group of birds or fishes. In this method the population of animals and the candidate solutions are known as swarm and particles, respectively^44^. The PSO algorithm is based on the collective behavior of individuals in a community (Fig. 2). This means that there are a number of individuals, namely particles here, searching for the best place as a target or output variable for prediction in the community. Every particle has its own velocity, and it is done its own search iteratively for finding the best place. The best place is known as the solution or the prediction of the output variable. During the searching process of each individual particle, finding the new place is affected by two factors; the former is the best experience of the particle until that iteration; the latter is the best experience among all particles together.

The optimal location found for a particle is recorded and called $pbest$. The best position finding by the whole particles is called $gbest$*.* Taking $pbest$*, gbest* and each particle’s velocity, the update rule for their location is as follows^45–47^:

12 $$V_{t + 1} = W_{t} \times V_{t} + C_{1} \times rand\left( { } \right) \times \left( {pbest - x_{t} } \right) + C_{2} \times rand\left( { } \right) \times \left( {gbest - x_{t} } \right),$$

13 $$x_{t + 1} = x_{t} + V_{t + 1},$$

where $W$ represents the inertia weight showing the impact of the fr velocity vector $\left( {V_{t} } \right)$ on the new vector, $C_{1}$ and $C_{2}$ denote the acceleration constants and $rand \left( { } \right)$ represents a random function in the range $\left[ {0, 1} \right]$ and $x_{t}$ denotes the present location of the pticle.

Table 1 summarizes the parameters of the PSO algorithms that are used in this study. The values of swarm size and inertia weight are adjusted in order the best intelligence to be achieved, while the inertia weight damping ratio, the personal and the global learning coefficients have been fixed in the model.

**Table 1:** Particle swarm optimization algorithm parameters.

**PSO algorithm parameters**	
Swarm size	60, 80, 100, 120
Inertia weight (0–1)	0.85, 0.9, 0.95, 1
Inertia weight damping ratio	0.99
Personal learning coefficient (0–2)	1
Global learning coefficient (0–2)	2

### Fuzzy inference system (FIS)

FIS is a fuzzy engine in terms of the concepts of fuzzy if–then rules, and fuzzy set theory. In this intelligence approach, if–then rules presented by Takagi and Sugeno are run^48^. In this study x coordination, y coordination and z coordination are taken to attain liquid phase velocity as output. The kth rule function is:

14 $$w_{k} = \mu_{xk} \left( X \right) \mu_{yk} \left( Y \right)\mu_{zk} \left( Z \right) .$$

The detailed procedure is reported elsewhere^17–19,48^.

The fuzzy set parameters are described in Table 2. Totally, there are 9477 data that are created by the CFD simulation. 76% of the generated data are learned and the prediction is done after 400 iterations. The fuzzy C-means clustering is adopted as the cluster type. The type of membership function is Gaussian. The number of clusters, the rules, and the output membership function all is 30.

**Table 2:** Fuzzy inference system parameters.

**Fuzzy inference system parameters**	
Percentage of data for training (P)	76%
Number of data	9477
Iteration	400
Data clustering method	FCM clustering
Membership function (MF)	Gaussmf
Number of cluster (NC)	30
Number of rules and output MFs	30

## Results and discussion

Two-phase air–water flow inside a bubble column type chemical reactor is simulated via CFD method. The air is injected into the bubble column filling with water^49^. For the first time in this study, the temperature of the air (127 °C) differs from the water (23 °C). As a result, there is not any thermal equilibrium between air and water. This requires the additional governing equation of energy for the CFD modeling. All governing equations (i.e. mass, momentum, energy, turbulence model) are considered in the Eulerian–Eulerian framework for the simulation of process. This means the equations are solved for each phase separately and coupled with each other in the source terms. Therefore, solving two-phase CFD models, the 3D modeling, considering the effect of turbulent flow imposes massive computational efforts. The PSO algorithm-based fuzzy inference system (PSOFIS) was selected for the CFD modeling simplification.

**Figure 3:**
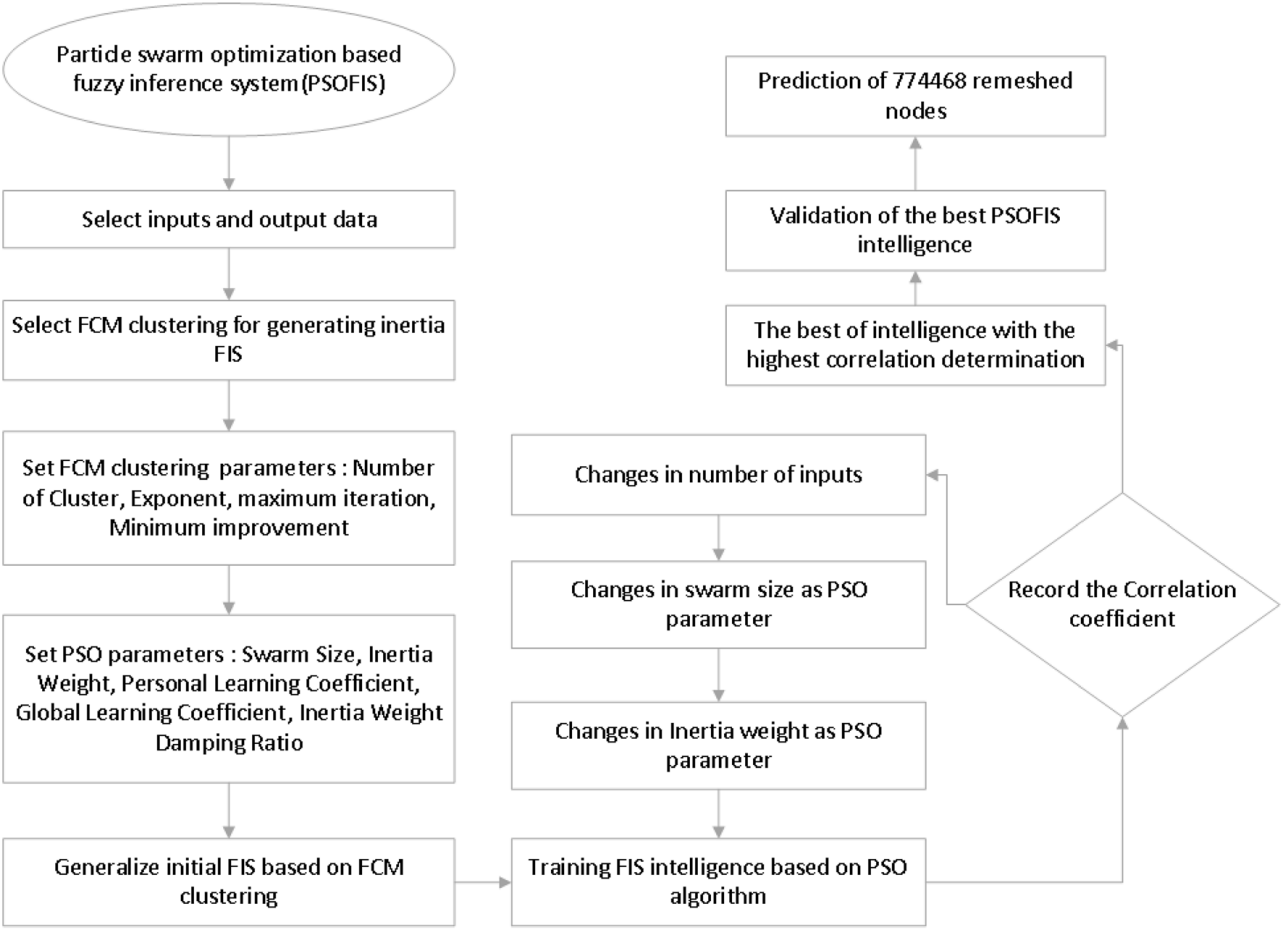
Schematics of combination of PSO algorithm and fuzzy inference system.

**Figure 4:**
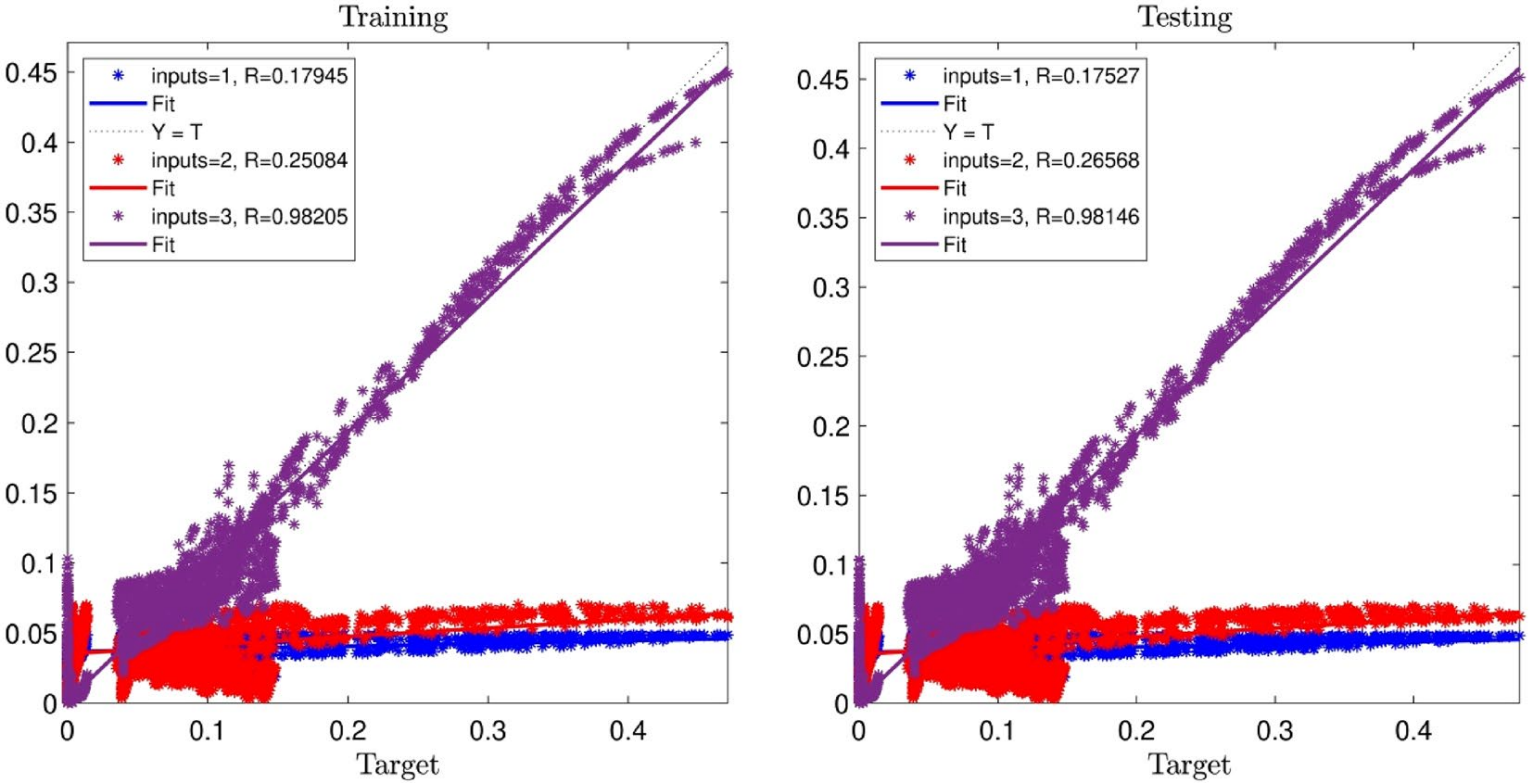
PSOFIS learning processes with considering changes in number of inputs.

Figure 3 illustrates the flowchart of the PSO algorithm designed for this study. PSOFIS learns the CFD data generated by the numerical simulations for the prediction of a specific variable as the output (target). In the present study, liquid phase (i.e. water) velocity is the output value. The x, y, and z coordinates of the nodes’ location of the water are the inputs. 76% of the whole CFD data (i.e. 9477 data) is trained by the PSOFIS, while the 100% CFD data are used in testing. The regression is adopted as an index for reaching the best intelligence. Different input numbers (i.e. 1, 2, and 3), swarm sizes (i.e. 60, 80, 100, and 120), and inertia weights (i.e. 0.85, 0.9, 0.95, and 1) are tested for achieving the best intelligence. Figure 4 shows the influence of input numbers on the regression number. As the number of input increases, the regression numbers go up for both training and testing. The best intelligence is achieved when the input number is equal to 3 (i.e. R = 0.98).

**Figure 5:**
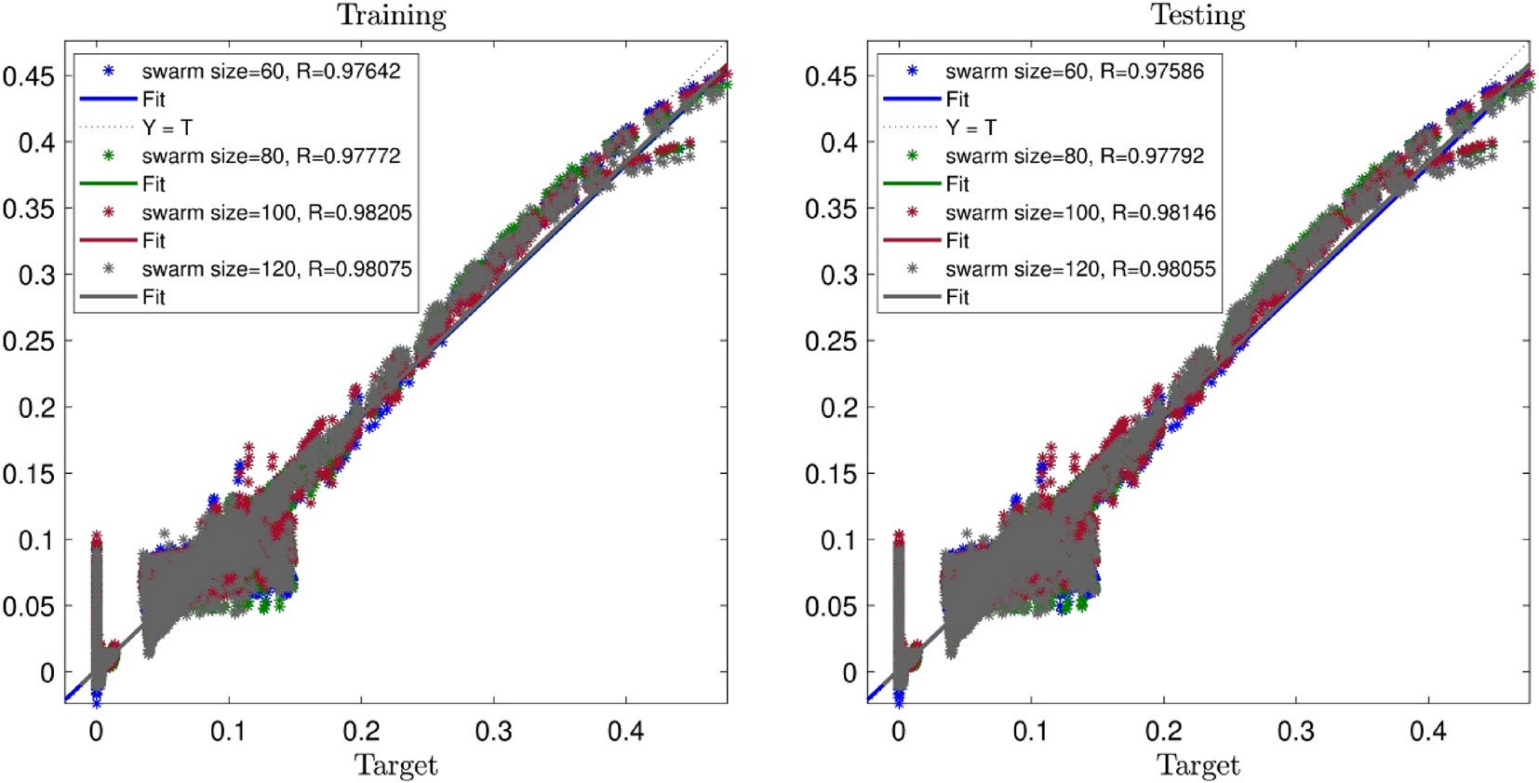
PSOFIS learning processes with considering changes in swarm size when number of inputs is 3.

A similar sensitivity test is done for the effect of swarm sizes, and inertia weights when the input number is 3. Figure 5 illustrates the PSOFIS learning processes with considering changes in swarm size when number of inputs is 3. According to this figure, by increasing swarm sizes from 60 to 100, the R value increases from 0.97 to 0.98. However, the further increases of swarm size to 120, no significant changes are seen in the value of R.

**Figure 6:**
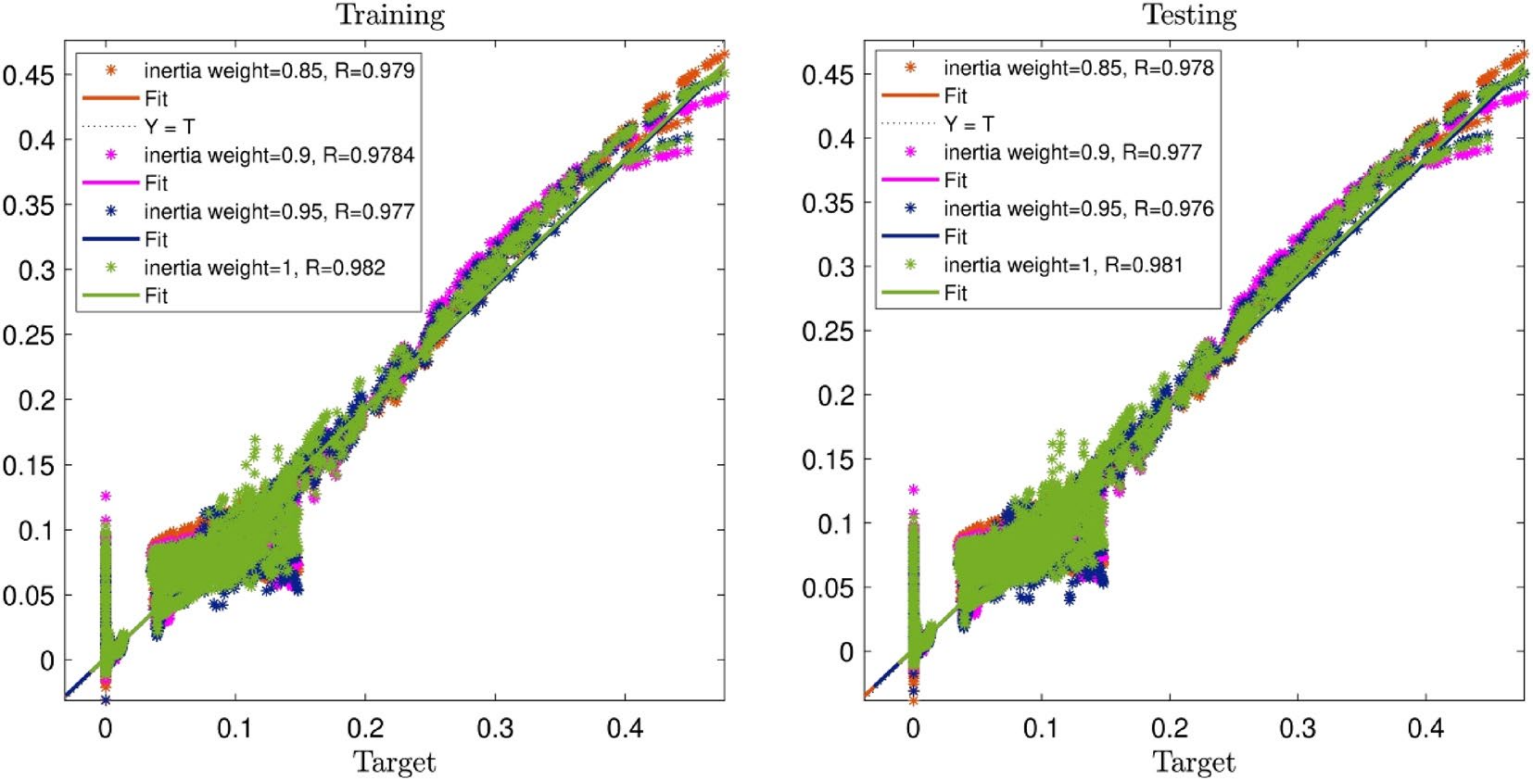
PSOFIS learning processes with considering changes in inertia weight when swarm size is 100 and number of inputs is 3.

Figure 6 depicts the PSOFIS learning processes with considering changes in inertia weight when swarm size is 100 and number of inputs is 3. Regarding this figure, rising inertia weights from 0.85 to 1, the R value increases from 0.97 to 0.98. Hence, the best intelligence can be obtained for the input number of 3, swarm size of 100, and inertia weight of 1.

**Figure 7:**
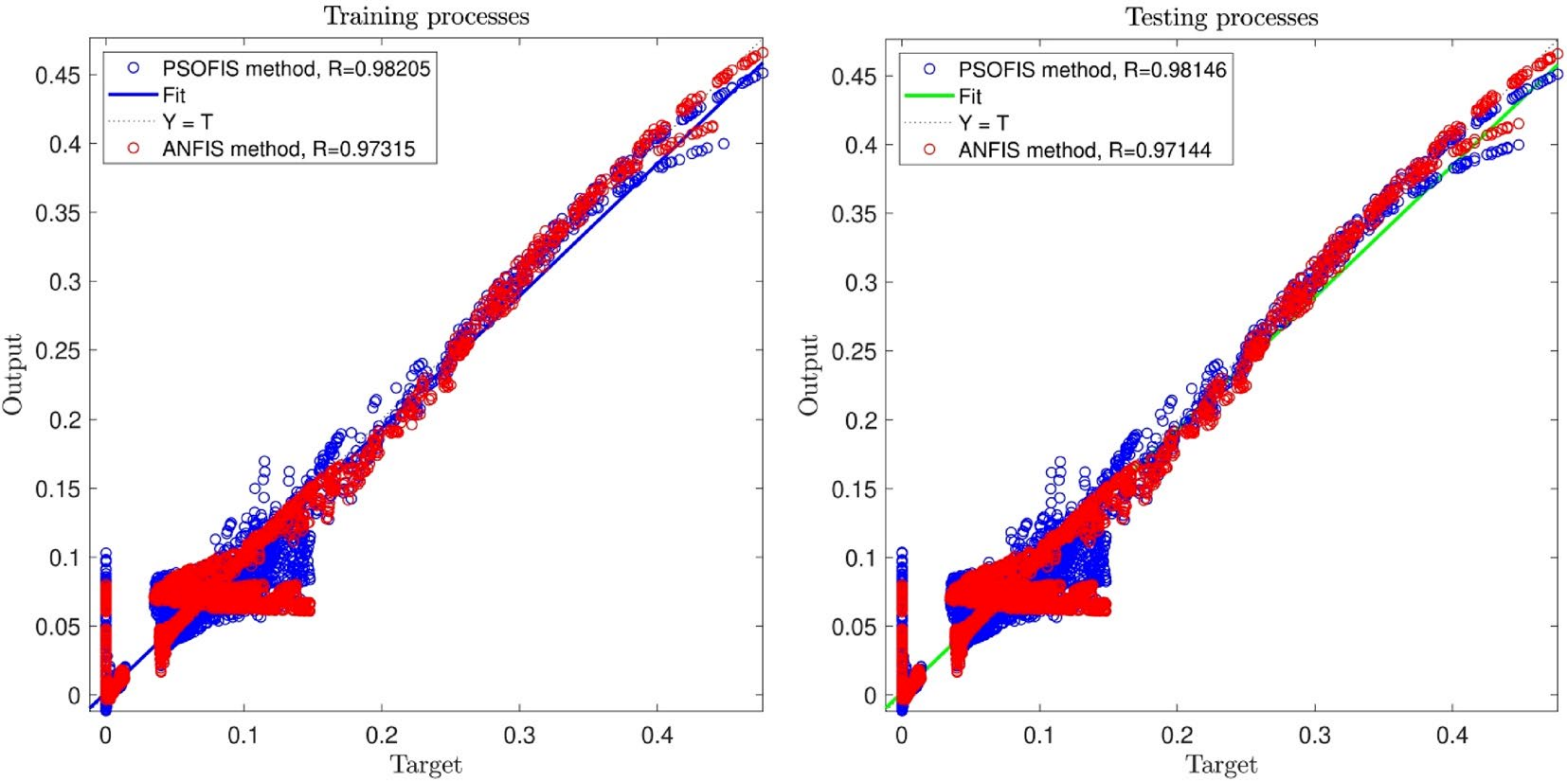
Comparison of the best result of PSOFIS and ANFIS methods.

**Figure 8:**
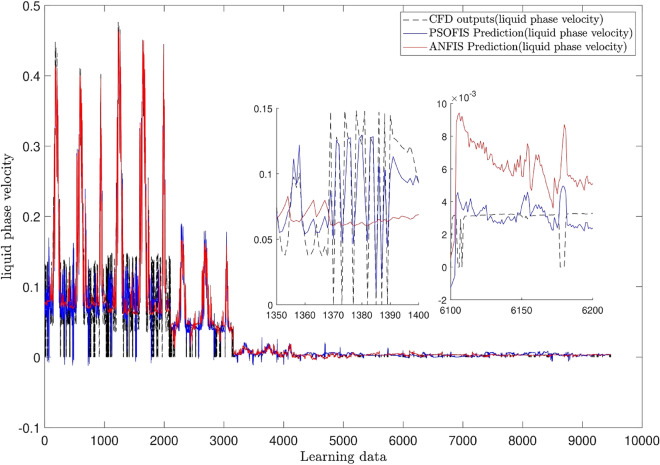
Pattern of liquid phase velocity in different methods.

For more validation, the integration of the adaptive network with the fuzzy inference system, calling ANFIS, is employed for learning the CFD outcomes for simulating of the water velocity inside the reactor^49^. The results of the PSOFIS are compared with the ANFIS. It should be noted that for the similar setup condition, all the fuzzy set parameters of the ANFIS are the same as the PSOFIS (referring to Table 2). According to Fig. 7, the R value of the PSOFIS (0.98) is a little more than that of the ANFIS (0.97). Figure 8 illustrates the pattern of the liquid phase velocity in both methods of ANFIS and PSOFIS. Magnifying the graphs, it is seen that the PSOFIS predictions follow the CFD results with more compatibility in comparison with the ANFIS.

**Figure 9:**
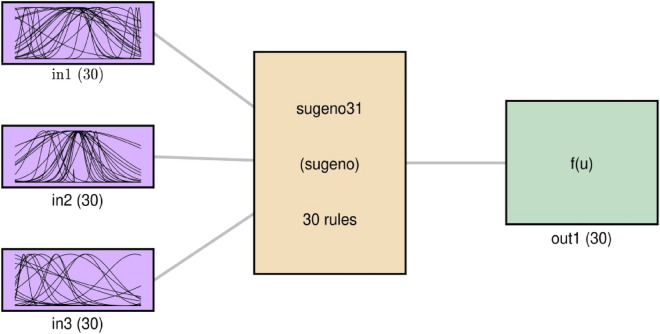
FIS structure using PSO algorithm as trainer in learning process.

The FIS structure based on PSO learning process is shown in Fig. 9 in a schematic way. The membership function is Gaussian as shown in input boxes on the right. The number of clusters in for each input, the number of rules in the hidden layer, and the number of membership functions for output are 30. The type of cluster is fuzzy semi clustering.

**Figure 10:**
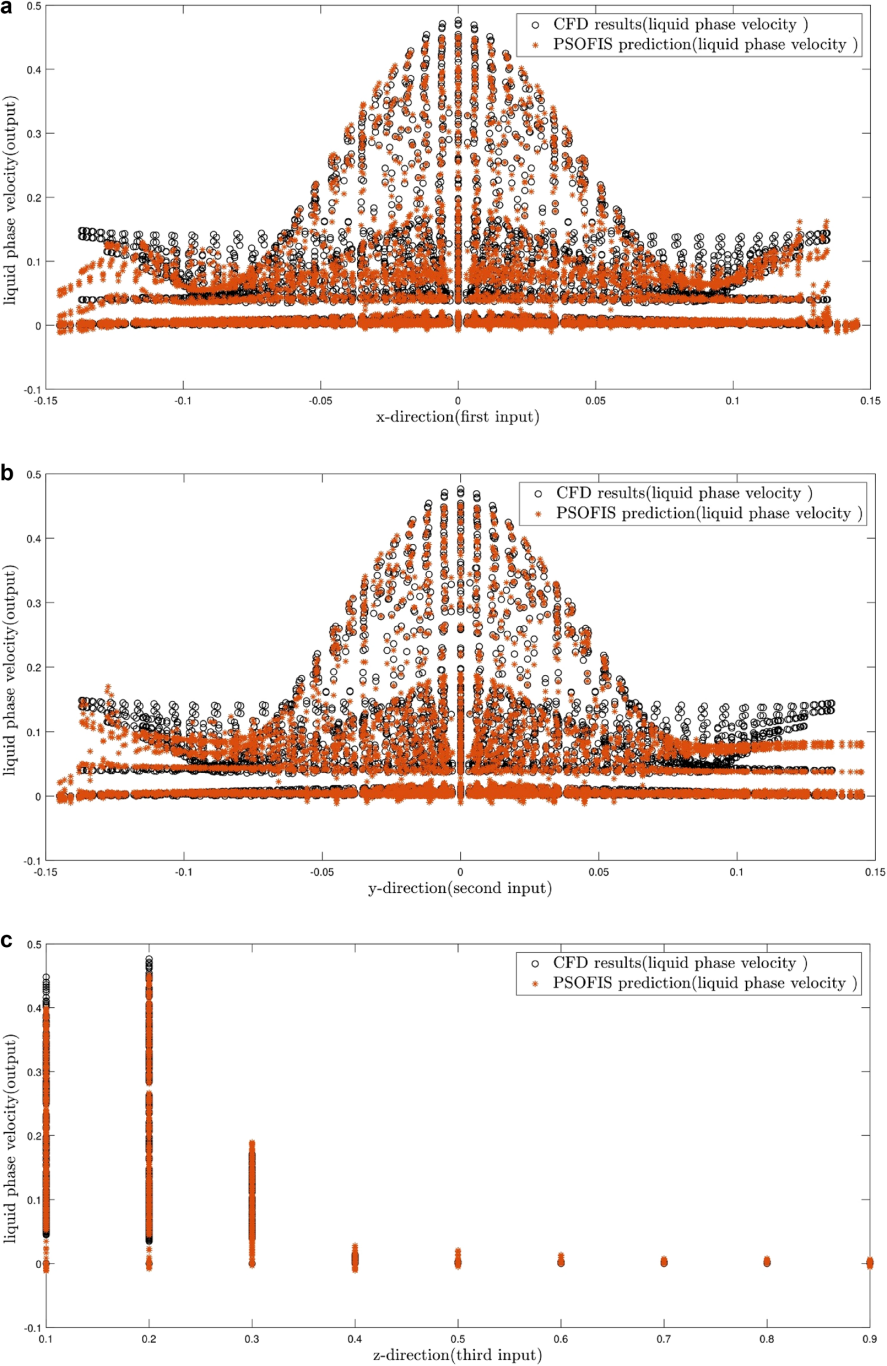
(**a**) Validation of PSOFIS learning process after achieving the highest PSOFIS intelligence based on first input. (**b**) Validation of PSOFIS learning process after achieving the highest PSOFIS intelligence based on second input. (**c**) Validation of PSOFIS learning process after achieving the highest PSOFIS intelligence based on third input.

Figures 10a–c illustrate the comparison between the CFD and the PSOFIS predictions of water velocity in each input. As seen, there is no conflict between the results of both methods. Since the results are for the time of 30 s, as to be expected, the water velocity is higher at lower heights of the column (i.e. z = 0.1 and 0.2 m) and the velocity is damped to reach zero at higher heights (i.e. z = 0.9 m).

**Figure 11:**
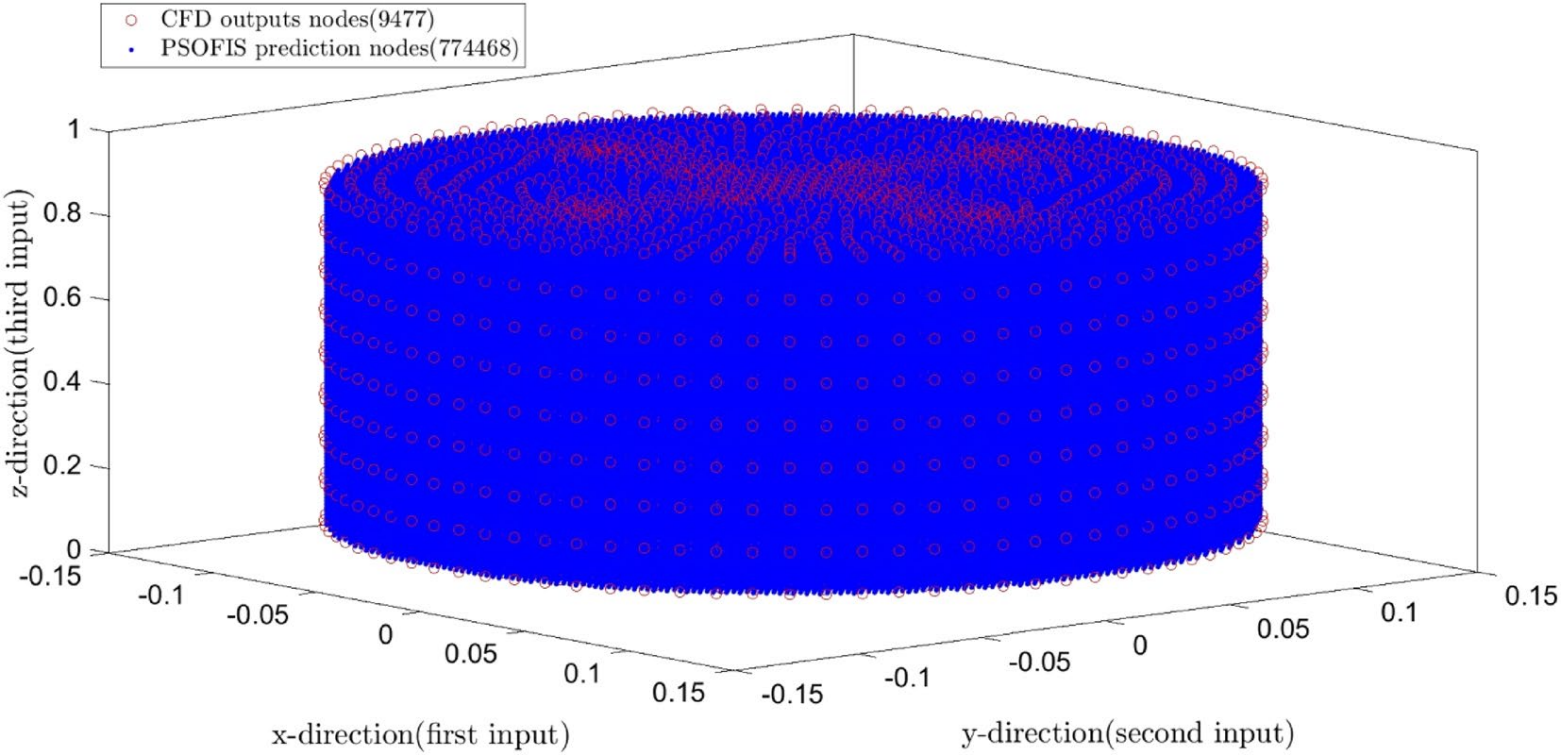
Remeshed domain from 9477 to 774,468.

After reaching the best intelligence, the water velocity can be found for more nodes in the column domain without mesh refinement in the CFD domain. In fact, the mesh refinement can be done in the PSOFIS with much less computational efforts. Figure 11 illustrates the increase of the mesh density from 9477 to 774,468 nodes using the PSOFIS method.

**Figure 12:**
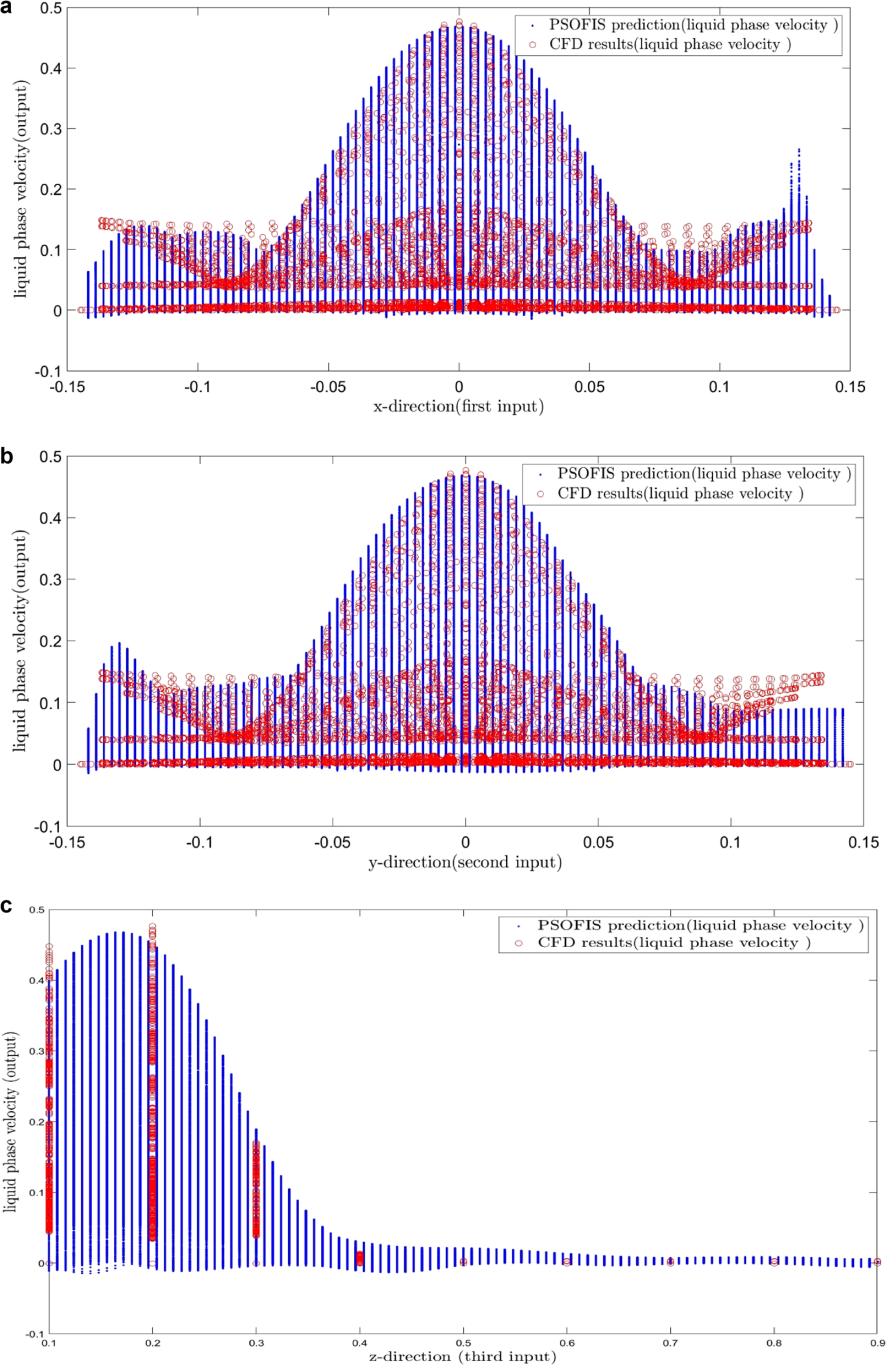
(**a**) Liquid phase velocity prediction of PSOFIS in 774,468 nodes based on first input. (**b**) Liquid phase velocity prediction of PSOFIS in 774,468 nodes based on second input. (**c**) Liquid phase velocity prediction of PSOFIS in 774,468 nodes based on third input.

The results of the PSOFIS predictions of the water velocity are depicted in Fig. 12. The new predictions of the PSOFIS method are in agreement again with the CFD results. Moreover, additional predictions of the water velocity in more nodes are seen in Fig. 12a–c.

## Conclusions

The two-phase flow of air–water inside a bubble column reactor with a non-equilibrium thermal condition between air and water was simulated by the CFD method. The hot air with a temperature of 127 °C was injected into the water column with a temperature of 23 °C. The Eulerian two-phase CFD model was implemented for turbulent flow inside the bubble column. The artificial intelligence algorithm and in specific the particle swarm optimization (PSO) algorithm-based fuzzy inference system (PSOFIS) was employed to help such complicated CFD modeling. A lot of computational cost, effort, and time are saved by the reduction of the number of CFD simulations. Once the best intelligence is achieved, no need for simulation anymore.

The PSOFIS was used to predict the water velocity at x, y, and z nodes positions in the column. The regression number was considered as an index for the best intelligence. The proper values of input numbers, swarm sizes, and inertia weights were investigated for the best intelligence. For more validation, the integration of the adaptive network with the fuzzy inference system (ANFIS) was used for learning the CFD data. The results of the PSOFIS were compared with the ANFIS for the same fuzzy set parameters.

At the best intelligence of the PSOFIS, the water velocity was found for additional nodes without mesh refinement in the CFD domain. In fact, the mesh refinement could be done in the PSOFIS with much less computational efforts.

The results of this study are summarized as follows:

- The best intelligence is found for the input number of 3, swarm size of 100, and inertia weight of 1 where the regression number is around 0.98.
- The PSOFIS predictions follow the CFD results with more compatibility in comparison with the ANFIS. The regression number (R) of the PSOFIS (0.98) was more than that of the ANFIS (0.97).
- Comparison between the CFD and the PSOFIS predictions of water velocity shows no conflict between the results of both methods.
- The prediction of the water velocity shows a logic trend by increasing the height of the column. As expected, the water velocity is higher at lower heights of column (i.e. z = 0.1 and 0.2 m) and the velocity is damped to reach zero at higher heights (i.e. z = 0.9 m).
- Increasing mesh density of the bubble column from 9477 to 774,468 by the PSOFIS method, the new prediction of the PSOFIS method covers all the CFD results with additional predictions of the water velocity in more nodes in the domain.
